# Measuring airway compliance of pulmonary fibrosis by endobronchial optical coherence elastography

**DOI:** 10.1371/journal.pone.0351119

**Published:** 2026-07-10

**Authors:** Hang Xu, Jian-yi Niu, Zi-qing Zhou, Li-ya Lu, Chun-li Tang, Yang-huan Chen, Shi-yue Li, Ye Gu, Yu Chen

**Affiliations:** 1 Department of Pulmonary and Critical Care Medicine, The First Affiliated Hospital of Guangzhou Medical University, National Center for Respiratory Medicine, National Clinical Research Center for Respiratory Disease, State Key Laboratory of Respiratory Disease, Guangzhou Institute of Respiratory Health, Guangzhou, Guangdong, China; 2 Department of Anesthesiology, The First Affiliated Hospital of Guangzhou Medical University, Guangzhou, Guangdong, China; 3 Department of Endoscopy Center, Shanghai Pulmonary Hospital, Tongji University School of Medicine, Shanghai, China; Kurume University School of Medicine: Kurume Daigaku Igakubu Daigakuin Igaku Kenkyuka, JAPAN

## Abstract

**Background:**

Optical coherence elastography derived from optical coherence tomography for measuring soft tissue and organ compliance, holds promise in respirology but remains largely exploratory.

**Methods:**

The airway lumen area (Ai) was measured by endobronchial optical coherence tomography in control subjects (n = 4), and pulmonary fibrosis (n = 8) while airway pressure (Paw) increased from 0–20 cm H_2_O. Airway compliance (AC) and airway specific compliance (ASC) were derived from the Paw vs. Ai curves. Evaluate correlations among Ai, AC, ASC, and lung function parameters.

**Results:**

Endobronchial optical coherence elastography (EB-OCE) was constructed by ASC, which could detect AC and ASC among 3^rd^ to 7^th^ generations of bronchi. Pulmonary fibrosis tended to exhibit lower ASC in 5^th^ to 7^th^ generations of bronchi compared to controls. The ASC-7 appeared to be positively correlated with FEV₁, FVC, TLC, VC, and DLCO.

**Conclusions:**

EB-OCE provides a novel approach to measure AC and extends the analysis to small airway. Pulmonary fibrosis appeared to show a heterogeneous reduction in AC across different bronchial generations compared to controls. A decline in ASC-7 was possibly associated with reduced lung function.

## Introduction

Optical coherence tomography (OCT) is among the most innovative and successfully translated imaging technologies [1]. OCT is a non-invasive optical analog to ultrasound with significantly higher resolution (<1 μm), which closely matches conventional histopathology. It has found applications in fields such as ophthalmology, cardiology, and respirology. In recent years, OCT has continued to evolve to address diverse clinical needs. A notable area of research is optical coherence elastography (OCE), which holds considerable potential for the supplemental assessment of soft tissue and organ compliance, extending its utility beyond morphological analysis [2]. OCE functions similarly to palpation by combining OCT with excitation equipment to monitor the deformation of soft tissues and organs under testing conditions. By measuring elasticity, it reflects pathophysiological changes, thereby supporting clinical decision-making [3,4]. In ophthalmology, OCE has been employed to evaluate the biomechanical properties of structures such as the cornea [5], lens [6], and sclera [7]. However, its application in respirology is still in an exploratory phase, presenting substantial potential for future development.

Williamson and colleagues [8] measured airway mechanical properties, including airway compliance (AC) and airway specific compliance (ASC), normalized to airway size from the 0 to the 5^th^ generation of bronchi in obstructive pulmonary diseases using anatomical aOCT. aOCT is an emerging light-based imaging technique with the unique capacity to directly proﬁle hollow organs such as the airways during bronchoscopy [9]. Bu and colleagues [10] extended this work by developing OCE based on aOCT in animal studies, enabling the visualization of AC. These studies provide valuable insights and reference points for advancing endobronchial OCE (EB-OCE). However, the focus of these investigations has been predominantly on large and medium airways, with limited attention to more peripheral airways.

Small airway, defined as diameter﹤2 mm [11], and comprising cartilage-free rings, are of particular interest as they may exhibit distinct and more discernible biomechanical properties compared to larger airways. The probes used in the aforementioned studies, with a diameter of 3 mm [9], are incapable of measuring small airway. Our group has previously utilized endobronchial optical coherence tomography (EB-OCT) with a 0.9 mm catheter to measure the airway inner luminal area (Ai) across the 3^rd^ to 9^th^ generations of bronchi [12]. And we have found that the small airway originates from the 7^th^ generation of bronchi [13]. However, the scanning was performed in an auto-pullback mode, with the entire process lasting three seconds.

Consequently, it was not possible to identify the precise respiratory phase during which airway images were captured in spontaneous breathing. Moreover, the pressure applied during image acquisition could not be determined. This limitation significantly hampers the accurate measurement of AC. We hypothesize that combining EB-OCT with mechanical ventilation could provide stable, controllable, and variable pressures as well as sufficient scanning time to detect Ai at different pressure states, enabling the measurement of AC *in vivo*.

To test this hypothesis, we employed EB-OCT to detect Ai across several generations of bronchi under varying airway pressures (Paw) induced by mechanical ventilation. AC and ASC were derived from the Paw vs. Ai curves, and EB-OCE was constructed based on ASC to enable visualization. Finally, we investigated whether AC differs between individuals with pulmonary fibrosis and control subjects.

## Methods

### Subjects

Twelve patients aged 27 and 69 years who required bronchoscopy for diagnosis at the 1^st^ affiliate hospital of Guangzhou Medical University between December 28, 2022, and December 28, 2023, were enrolled in this study. The primary inclusion criteria were pulmonary fibrosis or solitary pulmonary nodule as identified by HRCT. The primary exclusion criteria included respiratory infections within the preceding four weeks, other respiratory diseases such as COPD and asthma, or inability to tolerate an esophageal balloon. Patients diagnosed with solitary pulmonary nodules and normal lung function served as control subjects. The study protocol adhered to the Declaration of Helsinki and received approval from the Ethics Committee of the First Afﬁliated Hospital of Guangzhou Medical University. Written informed consent was obtained from all participants. This study was registered with the registration number of NCT05692362.

### Pulmonary function tests

Pulmonary function tests (PFTs) were carried out according to American Thoracic Society [14], using an automated Vmax V6200 system (Sensor Medics). Parameters measured included FVC, FEV_1_, FEV_1_/FVC, TLC, VC, and DLCO. The DLCO results were adjusted for hemoglobin levels for each individual test.

### Bronchoscopy

An esophageal manometry balloon was placed into esophagus for respiratory mechanical monitoring [15]. Respiratory mechanical examination and OCT scanning were performed under deep sedation [intravenous propofol (1–3 μg/mL) and remifentanil (2–4 ng/mL)], muscle relaxation (MR) [intravenous rocuronium bromide (0.6 mg/kg)], and mechanical ventilation via endotracheal tube. The degree of muscle relaxants was monitored by four responses occur after train-of-four (TOF). In addition to standard anesthetic monitoring, physiological parameters included airway pressure at the proximal end of the endotracheal tube (Pm), which corresponded to airway pressure (Paw).

### Endobronchial optical coherence tomography

An 0.9 mm diameter OCT catheter with 180 Hz rotary frame rate and 1.8 cm/s pullback rate was inserted to the RB8 segment (anterior basal segment of the right lower lobe) by using a flexible bronchoscope (B260F, Olympus, Tokyo, Japan) as previously reported [12]. In each scanning, a total of 540 consecutive images were obtained from the 3^rd^ to 9^th^ generation of bronchi and were stored to the Lightlabs C7XR (St. Jude Medical, St. Paul, MN, USA) OCT system, axial profiles were digitized in each scan position to create a three-dimensional image of the airway. Ai of each generation was analyzed by using deep learning system as previously reported [16].

### Scanning protocol

**Fig 1:**
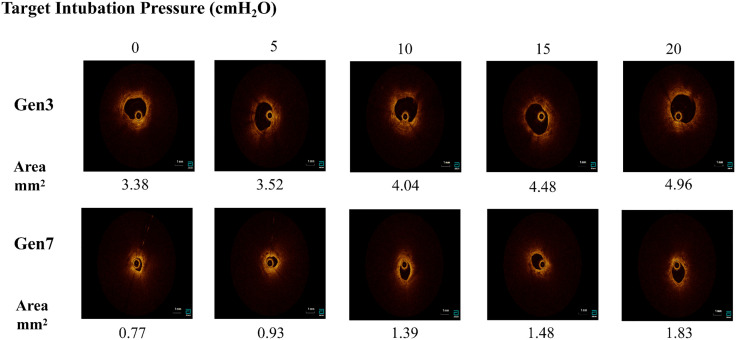
The Ai of 3rd and 7th generation of bronchi increased with pressure.

Paw was incrementally increased from 0 to 20 cm H_2_O in 5 cm H_2_O intervals. At each Paw level (0, 5, 10, 15, and 20 cm H_2_O), OCT scanning was conducted at least three times (Fig 1). A more detailed explanation of the study protocol is provided in the online supplement.

#### Data analysis and color rendering.

**Fig 2:**
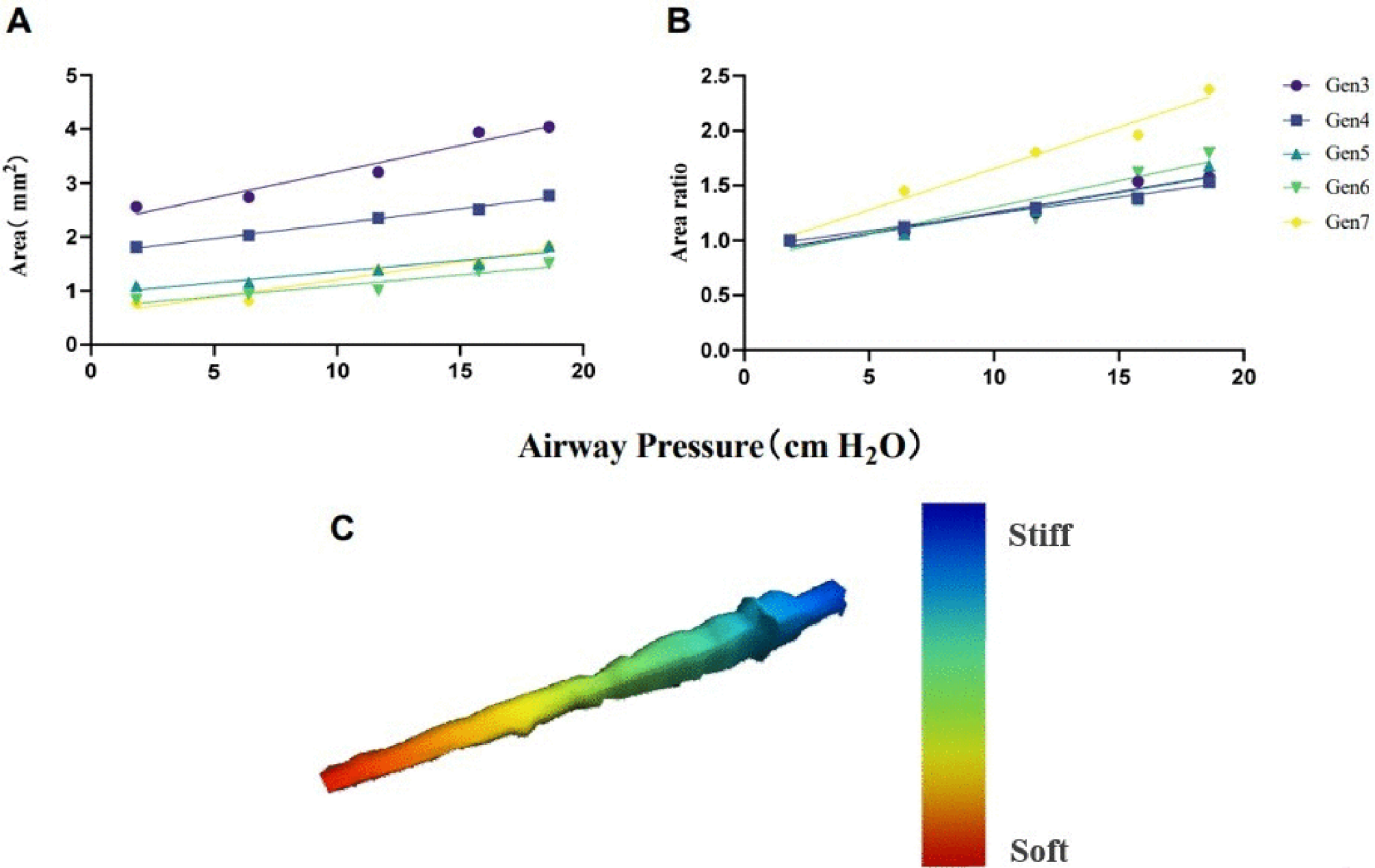
Airway compliance and specific compliance calculations and elastography renderings.

AC and ASC were measured from the 3^rd^ to 7th generations of bronchi. The relationship between Ai and Paw was represented as a line (Fig 2). AC was defined as the slope of the ratio between Paw and Ai. ASC was also determined (AC/Ai, Ai at the 0 cm H_2_O) to normalize measurements for airway size. The correlations of these parameters with airway generations were analyzed.

The three-dimensional airway structure was rendered with color coding based on the ASC of each generation using MAYA software (Fig 2C). Colors ranged from blue to red depending on the magnitude of ratio (S1 Table).

### Statistical analysis

Statistical analyses were conducted using SPSS 20.0 (SPSS Inc, USA), GraphPad Prism 9.0 (GraphPad Inc, USA), and RStudio. Demographic data, EB-OCT measurements, and respiratory mechanics were compared using either the A-NOVA test or the Kruskal-Wallis test, depending on data distribution. The correlations between Ai, AC, ASC, and lung function parameters were assessed using the Spearman test. A bilateral p value <0.05 was considered statistically significant.

## Results

A total of 12 subjects were included, 4 in the control group and 8 in the pulmonary fibrosis group, half of each according to the presence or absence of traction bronchiectasis. No significant differences were observed in the demographic characteristics among the three groups (all p > 0.05). Patients with pulmonary fibrosis, both with and without traction bronchiectasis, exhibited lower FVC, FVC%, DLCO, and DLCO% compared to control subjects (all p < 0.05). Additionally, patients with pulmonary fibrosis and traction bronchiectasis had a greater Ai at the 5^th^ generation of bronchi compared to controls (p < 0.05). No significant differences were found in the other Ai parameters measured by EB-OCT among the three groups (all p > 0.05) (Table 1).

**Table 1:** Clinical Data.

	Control (n = 4)	Pulmonary fibrosis without traction bronchiectasis(n = 4)	Pulmonary fibrosis with traction bronchiectasis (n = 4)
**Sex (M/F)**	2/2	1/3	3/1
**Age (yrs)**	45 ± 19.8	58.25 ± 8.55	56.75 ± 15.89
**BMI (kg/m** ^**2**^ **)**	22.55 ± 3.55	20.93 ± 2.03	20.18 ± 2.19
**Others lung diseases**	0	0	0
**Smoker history(Y/N)**	0/4	1/3	3/4
**Lung function**			
FVC(L)	3.60 ± 0.45	2.14 ± 0.94*	1.73 ± 0.08*
FVC %	99.42 ± 21.22	67.27 ± 10.70*	65.19 ± 22.99*
FEV_1_(L)	2.84 ± 0.44	1.94 ± 0.81	1.56 ± 0.08
FEV_1_%	94.61 ± 23.39	73.92 ± 12.40	71.46 ± 15.77
FEV_1_/FVC	0.79 ± 0.12	0.95 ± 0.10	0.89 ± 0.09
TLC(L)	4.80 ± 0.69	3.37 ± 1.47	2.90 ± 0.26
VC(L)	3.26 ± 0.43	2.11 ± 1.03	1.63 ± 0.06
DLCO (mmol/min/kPa)	7.78 ± 0.44	3.97 ± 1.74*	2.89 ± 0.27*
DLCO %	89.81 ± 5.72	49.42 ± 17.38*	40.40 ± 8.91*
**Ai(mm** ^**2**^ **)**			
Gen3	4.57 ± 1.23	5.16 ± 1.54	6.36 ± 3.40
Gen4	3.07 ± 1.70	3.09 ± 0.63	6.39 ± 1.98
Gen5	1.44 ± 0.50	2.05 ± 0.65	4.69 ± 1.89*
Gen6	0.93 ± 0.42	1.65 ± 0.81	4.17 ± 4.49
Gen7	0.83 ± 0.24	1.44 ± 0.93	3.53 ± 3.78

**Abbreviations:** 2 subjects in pulmonary fibrosis with traction bronchiectasis were unable to tolerate pulmonary function. FVC: Forced Vital Capacity; FVC%: Percentage of expected value measured through Forced Vital Capacity; FEV_1_: Forced Expiratory Volume in the first second; FEV_1_%: Percentage of expected value measured in the first second of Forced Expiratory Volume; FEV_1_/FVC: Percentage of Forced Expiratory Volume to Vital Capacity in the first second. TLC: Total Lung Capacity. VC: Vital Capacity. DLCO: Diffusing capacity of the lung for carbon monoxide (CO). DLCO%: Percentage of expected value measured in the diffusing capacity of the lung for carbon monoxide. The data were presented as mean value with associated standard deviation. Pairwise comparison of patients in the three groups based on their gender and smoking history was performed using the Chi-square test. Kruskal-Wallis test was employed for comparing Ai from Gen4 to Gen7 among the three groups. The ANOVA test was employed for comparing patients among the remaining three groups.

All subjects successfully underwent EB-OCE scanning during intubation and mechanical ventilation. The mean duration of each EB-OCE scan was 25.3 minutes (range: 21–32 minutes). No adverse events or complications occurred. The generation-precision detection range of AC was extended to the 7^th^ generation of bronchi by EB-OCE.

Across all 12 subjects, no significant differences were identified in AC and ASC across the measured bronchial generations. However, after grouping, ASC-7 (0.068 ± 0.012) was significantly higher than ASC-3 (0.028 ± 0.010) in the control group (p = 0.023). In pulmonary fibrosis with traction bronchiectasis, AC-5 (0.119 ± 0.062) was higher than in controls (0.061 ± 0.016, p = 0.072). Additionally, ASC-7 in pulmonary fibrosis with traction bronchiectasis (0.035 ± 0.006) was significantly lower compared to controls (0.068 ± 0.012, p = 0.008) and pulmonary fibrosis without traction bronchiectasis (0.056 ± 0.011, p = 0.075). No significant differences in AC or ASC were observed in the remaining generations (Table 2).

**Table 2:** Airway Compliance and Specific Compliance Between generations and groups.

Airway Generation	Control		Pulmonary fibrosis without traction bronchiectasis		Pulmonary fibrosis with traction bronchiectasis	
	AC	ASC	AC	ASC	AC	ASC
**Gen3**	0.151 ± 0.097	0.028 ± 0.010	0.189 ± 0.064	0.037 ± 0.002	0.223 ± 0.229	0.029 ± 0.014
**Gen4**	0.102 ± 0.060	0.034 ± 0.003	0.116 ± 0.026	0.039 ± 0.011	0.183 ± 0.158	0.026 ± 0.016
**Gen5**	0.061 ± 0.014	0.041 ± 0.002	0.080 ± 0.024	0.040 ± 0.002	0.119 ± 0.054	0.027 ± 0.020
**Gen6**	0.040 ± 0.011	0.050 ± 0.011	0.074 ± 0.027	0.049 ± 0.011	0.173 ± 0.191	0.040 ± 0.002
**Gen7**	0.047 ± 0.012	0.068 ± 0.012^†^	0.072 ± 0.031	0.056 ± 0.011	0.1055 ± 0.093	0.035 ± 0.006^*^

The AC and ASC of 4^th^ generation of bronchi failed to meet the criteria for a normal distribution, the Kruskal-Wallis test was utilized for group comparison. Other data sets exhibited a normal distribution, the A-NOVA test was employed for comparing data across multiple groups.

†: The data of the group were significantly different from the 3^rd^ generation of bronchi in control, p < 0.05.

*: The data of the group were significantly different from control individuals, p < 0.05.

**Fig 3:**
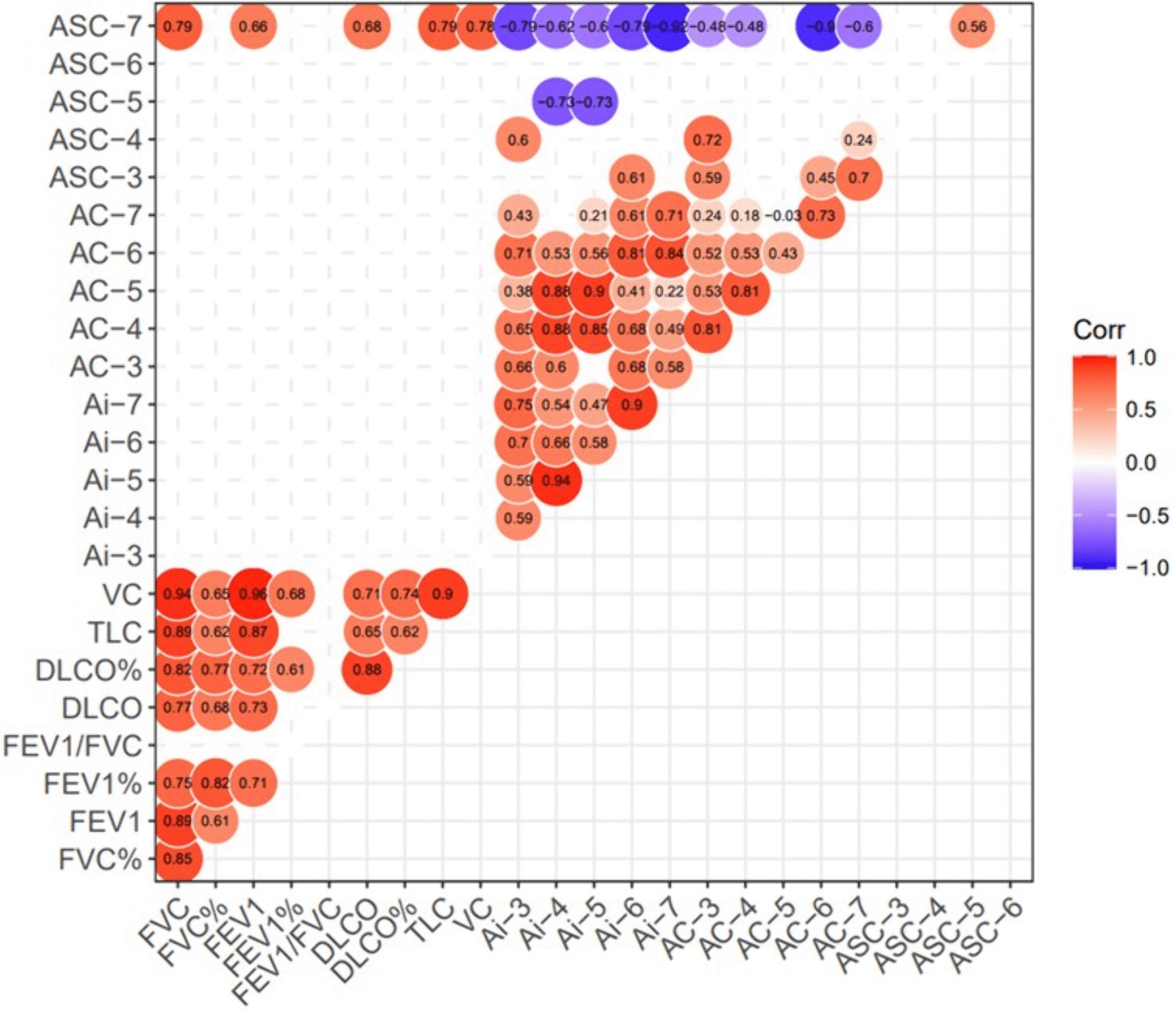
The correlation between the Ai, AC, ASC, and lung function.

A positive correlation was found between ASC-7 and pulmonary function parameters, including FEV_1_, FVC, TLC, VC, and DLCO (all p < 0.05). No significant correlations were observed between other airway biomechanical parameters and lung function (all p > 0.05) (Fig 3).

## Discussion

This study represents the first clinically applicable use of endobronchial optical coherence elastography (EB-OCE) to measure airway compliance from the 3^rd^ to the 7th generation of bronchi *in vivo*. We successfully extended the measurement of airway compliance to small airways in humans. Notably, distal ASC in control subjects was higher than in patients with pulmonary fibrosis, with the lowest distal ASC observed in patients with traction bronchiectasis. Additionally, ASC-7 was appeared to be positively correlated with key pulmonary function parameters, including FEV1, FVC, TLC, VC, and DLCO.

Compared to previous studies [8,10], this research employed several innovative methodologies to precisely measure small airway compliance. First, we utilized EB-OCT with a 0.9 mm catheter and a bronchoscopy navigation system to achieve accurate positioning and assess changes in airway dimensions across all bronchial generations [12]. Second, general anesthesia with endotracheal intubation and mechanical ventilation was implemented to ensure stable, controllable, and variable pressure during the measurement of airway morphological parameters. Additionally, we compared the factors that may affect the measurements. P_L_ (defined as Paw minus Ppl) is widely considered the true“lung-distending”pressure for assessing lung compliance [15] and has been used to measure the airway mechanical properties [8]. Ppl is surrogated by esophageal pressure (Peso), which requires patients to swallow esophageal ballon autonomously and uncomfortably [17]. Paw is also used for measuring airway mechanical properties during mechanical ventilation, with positive pressure ventilation titrated based on Paw to approximate P_L_ in sedated subjects [15]. However, the suitability of Paw as a surrogate for P_L_ in airway compliance measurement has been underexplored. To clarify the difference between the measurements by P_L_ and Paw. We monitored and compared the above respiratory indicators. We found the absolute values of P_L_ and Paw showed minimal difference, but the difference between ∆P_L_ and ∆Paw was negligible (S2 Table). AC is determined by the difference in area change compared to the change in pressure, the change in values (∆P_L_ and ∆Paw) is more critical than their absolute values. Therefore, we used Paw for measurement in this study. Previous studies have reported the muscle relaxants could increase airway resistance in animal model [18,19], yet their impact on airway compliance in humans is poorly understood. To figure out the effects of airway compliance with muscle relaxant drugs, all EB-OCE scans were performed before and after muscle relaxant. We found muscle relaxants did not significantly affect airway compliance (S3 Table). Previous studies have attributed airway resistance increases to histamine release caused by certain relaxants, such as mivacurium [18]. In contrast, we used rocuronium, which does not induce histamine release [20], this may be the reason why it has no significant effect on airway compliance. Considering muscle relaxant drugs are generally administered prior to intubation, the compliance indicators mentioned in our manuscript are all post-muscle relaxation data.

Research has shown that small airway specific compliance is significantly greater than that of larger airways in mice [21]. In humans, smaller-diameter airways exhibit a greater relative degree of expansion on CT [22], but the resolution limitations of CT prevent accurate measurement of small airways. In our study, ASC-7 (0.068 ± 0.012) was significantly higher than ASC-3 (0.028 ± 0.010) in control subjects (p < 0.05), indicating that small airways exhibit greater compliance compared to large airways in healthy humans. However, this pattern was not observed in pulmonary fibrosis, where fibrosis-induced reductions in ASC were evident in small airways. Previous studies have demonstrated decreased compliance of the entire airway tree [23] reduced global peripheral compliance in pulmonary fibrosis using oscillometry [24,25]. In the current study, we observed varying degrees of decline in ASC across different airway generations in pulmonary fibrosis, with more pronounced reductions in small airways. This significant decrease in small airway specific compliance may contribute to the observed changes in overall airway compliance.

It is important to note that accumulating evidence indicates small airways in pulmonary fibrosis undergo significant changes by histology or Micro-CT [26–28]. Harri et al. [29] found that small airways in early idiopathic pulmonary fibrosis (IPF) demonstrate significant bronchiolar loss by EB-OCT *in vivo*, and that small airway lumen stereology showed IPF-affected airways to be significantly larger, more distorted, and more irregular than those less affected by IPF. In addition, this team has developed Polarization-Sensitive‑EB‑OCT to assess fibrosis severity *in vivo* [30], will aid research on small airway structural alterations in pulmonary fibrosis. Our study provides a complementary contribution to small airway pathology in pulmonary fibrosis through functional assessment by EB-OCE *in vivo*. These studies indicate that EB-OCT and its derived technologies will play growing role in the study of small airway pathology in pulmonary fibrosis. Furthermore, we investigated the relationship between airway compliance and lung function. ASC-7 in our study was positively correlated with FEV_1_, FVC, TLC, VC, and DLCO. The correlation between ASC-7, FEV_1_, and FVC suggests that decreased ASC leads to insufficient airway dilation over time, impairing airflow dynamics. The correlations with TLC and VC imply that ASC, as part of overall lung compliance, may also influence lung capacity. Additionally, the positive correlation of ASC-7 with DLCO highlights the role of alveoli, which act as springs contributing to airway compliance [21,31,32], and the alveolar fibrosis not only affects alveolar function itself, but also stiffens the airway. The relationship between ASC and lung function suggests that it may be used in the variety of respiratory diseases.

In this study, we developed a clinically applicable EB-OCE capable of measuring airway compliance and explored its application in pulmonary fibrosis. In the future, this method could be extended to study a wide range of respiratory diseases involving the airways, offering an effective tool for assessing biomechanical changes, particularly in small airway diseases. However, our research has some limitations. First, the farthest generation of bronchi where airway compliance could be accurately measured was the 7^th^. Although the 9^th^ generation of bronchi could be detected at lower pressures (e.g., 0 or 5 cmH_2_O), it often became undetectable at higher pressures (e.g., 15 or 20 cmH_2_O). Second, the requirement for mechanical ventilation during measurements will impose constraints on clinical applications. In the present study, several methodological considerations—including the necessity of mechanical ventilation and the decision regarding the use of P_L_ and MR—resulted in a limited sample size, which in turn led to considerable within-group variability. Accordingly, our study primarily establishes and optimizes the EB-OCE methodology. The results suggest a trend toward better compliance in distal airways compared with proximal airways, as well as reduced compliance in pulmonary fibrosis relative to healthy controls. However, owing to the limited cohort and the exploratory nature of the analyses, false-positive results cannot be excluded. Therefore, our findings are best interpreted as showing a tendency toward heterogeneous airway stiffening in pulmonary fibrosis, along with a potential link between ASC‑7 and pulmonary function, rather than establishing firm conclusions. The study was not designed, nor powered, to define normative or disease-specific cut-offs for airway compliance. In future studies conducted under the simplified methodology, larger sample sizes will be needed to address inter-group variability and to clarify the relationship between compliance and pulmonary compliance, while self-controlled designs may be considered to assess longitudinal changes in airway compliance. Additionally, the role of airway wall composition in affecting AC has not been fully examined. While we observed trends between ASC-7 and the distribution of smooth muscle and elastic fibers, these differences were not statistically significant between groups (S5–S6 Figs). Finally, the interplay between AC and lung compliance, as well as the impact of AC changes on lung function, require further investigation.

## Conclusion

We developed EB-OCE, a novel tool capable of measuring airway compliance from the 3^rd^ to 7^th^ generations of bronchi *in vivo*. Our findings suggest that small airway compliance may differ in pulmonary fibrosis relative to normal subjects and is possibly associated with lung function.

## Supporting information

S1 Fig Measuring airway compliance by EB-OCT. (TIF)

S2 Fig Respiratory mechanics monitoring chart. (TIF)

S3 Fig Muscle relaxation monitoring chart displaying real-time recordings of the degree of muscle relaxation. (TIF)

S4 Fig Bronchoscopy fixation bracket. (TIF)

S5 Fig EVG and α-SMA immunohistochemical staining of the 7^th^ airway mucosa. (TIF)

S6 Fig Comparison of the content of Generation 7 airway mucosal biopsy tissue. (TIF)

S1 Table Airway Specific Compliance And Color. (TIF)

S2 Table The absolute and change value of Paw, Peso and P_L_. (TIF)

S3 Table AC and ASC Before and After Muscle relaxation. (TIF)

S1 File Supplement Manuscript. (DOCX)
