# Supplementary material for: Measuring airway compliance of pulmonary fibrosis by endobronchial optical coherence elastography
Source: PLoS One. 2026 Jul 10;21(7):e0351119. doi: 10.1371/journal.pone.0351119 (PMC13354069; doi:10.1371/journal.pone.0351119)
Supplement: S1 File — (DOCX) [file pone.0351119.s010.docx]

# Measuring Airway Compliance of Pulmonary Fibrosis By Endobronchial Optical Coherence Elastography

Hang Xu, Jian-yi Niu, Zi-qing Zhou, Li-ya Lu, Chun-li Tang, Shi-yue Li, Yu Chen

Online data supplement

# METHODS：

## EB-OCT:

An interventional pulmonary professional physician performs bronchoscopy and EB-OCT (see S1 Fig.A); due to the need for repeated EB-OCT scans at the same site, bronchoscopy fixation brackets are utilized for stabilization to prevent any artificial movement of the OCT catheter position. These brackets feature multiple flexible joints to accommodate the various bronchoscopy implementations required (see S4 Fig). An anesthesiologist administers preoperative general anesthesia and intraoperative muscle relaxation (see S1 Fig.B) and determines the depth of anesthesia and extent of muscle relaxation based on the Train-of-Four (TOF) detected by muscle relaxation monitoring (Bene Vision N15, Mindray, Shenzhen) (see S3 Fig). A bronchoscopy operation assistant guides navigation equipment (LungPoint VBN, Bronchus, Hangzhou) (see S1 Fig.C). A respiratory mechanics specialist physician utilizes and monitors respiratory mechanics equipment (PowerLab 16-channel electrophysiology recorder, AD Instruments, Australia) that enables real-time recording of Pm (mouth pressure) which Equivalent to Paw (airway pressure) and Peso (esophageal pressure). The pressure data is synchronized with OCT images, and P_L_ (transpulmonary pressure) is calculated from the Pm and Peso measurements. (see S1 Fig.D).

## Bronchial Biopsy:

An interventional pulmonary professional physician performed bronchial biopsy on 7^th^ airway, obtaining three specimens at once. The biopsy samples were then stained using EVG and a-SMA scarlet staining to evaluate the presence of elastic fibers and smooth muscle in the airways. Unacceptable specimens were excluded, and the percentage of specific staining in tissue area was determined by averaging measurements using ImageJ (see Figures 1 and 2) for intergroup comparison. The exclusion and evaluation methodology remains consistent with previous papers^S1^. The findings indicated a noticeable downward trend in the content of elastic fibers (see S5 Fig.A) and smooth muscle (see S5 Fig.B), although there were no significant differences observed among the three groups.

# REFERENCES

S1. Luo, Y.L., et al., A clinical and canine experimental study in small-airway response to bronchial thermoplasty: Role of the neuronal effect. Allergol Int, 2022. 71(1): p. 66-72.
